# Supplementary material for: Radiotherapy Combined with PD-1 Inhibition Increases NK Cell Cytotoxicity towards Nasopharyngeal Carcinoma Cells
Source: Cells. 2021 Sep 17;10(9):2458. doi: 10.3390/cells10092458 (PMC8470143; doi:10.3390/cells10092458)
Supplement: Supplementary file 1 [file cells-10-02458-s001.zip › Supplementary figure 1.pptx]

## Slide 1
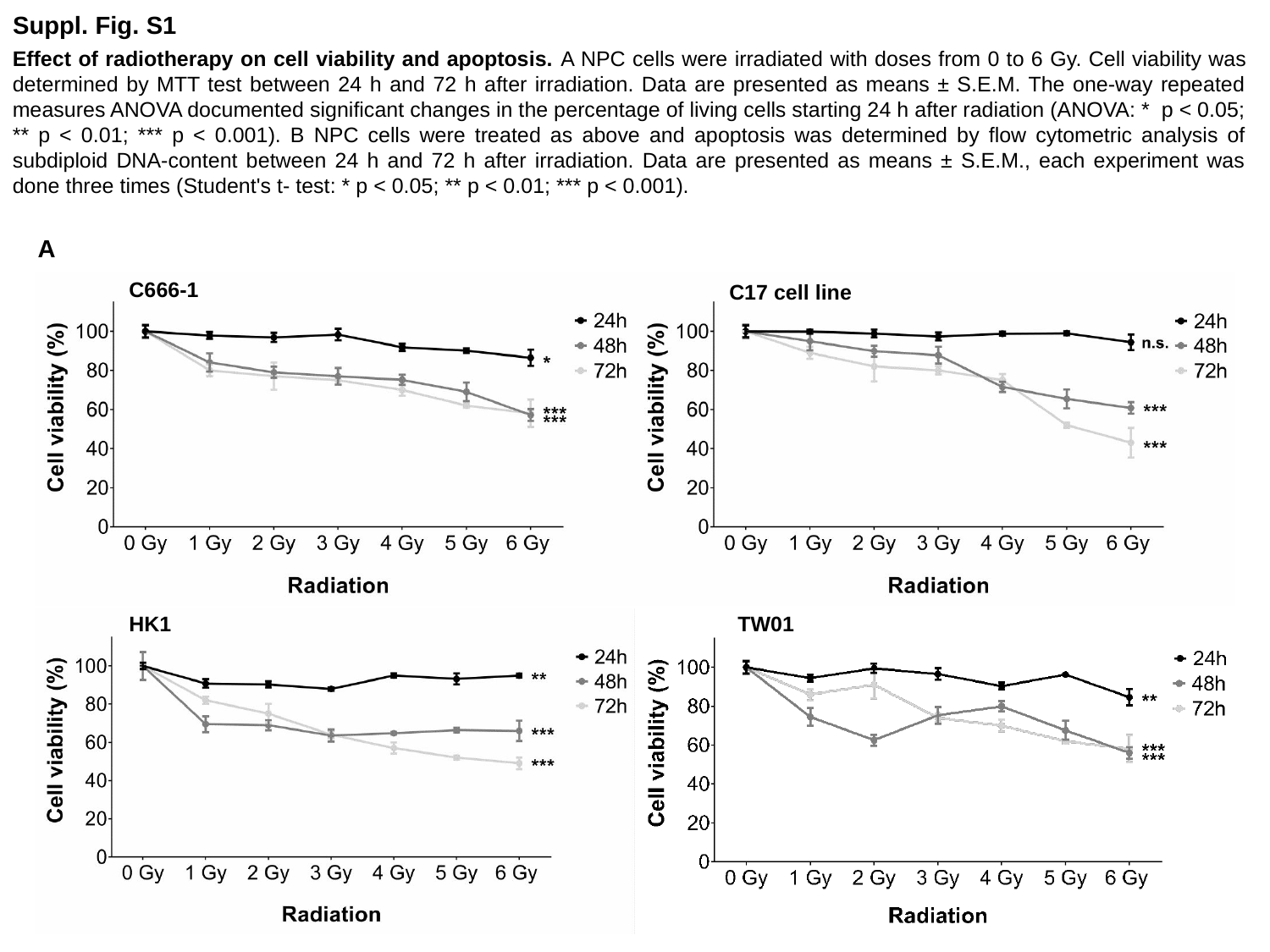

Suppl. Fig. S1
Effect of radiotherapy on cell viability and apoptosis. A NPC cells were irradiated with doses from 0 to 6 Gy. Cell viability was determined by MTT test between 24 h and 72 h after irradiation. Data are presented as means ± S.E.M. The one-way repeated measures ANOVA documented significant changes in the percentage of living cells starting 24 h after radiation (ANOVA: * p < 0.05; ** p < 0.01; *** p < 0.001). B NPC cells were treated as above and apoptosis was determined by flow cytometric analysis of subdiploid DNA-content between 24 h and 72 h after irradiation. Data are presented as means ± S.E.M., each experiment was done three times (Student's t- test: * p < 0.05; ** p < 0.01; *** p < 0.001).
A
C666-1
C17 cell line
TW01
HK1

## Slide 2
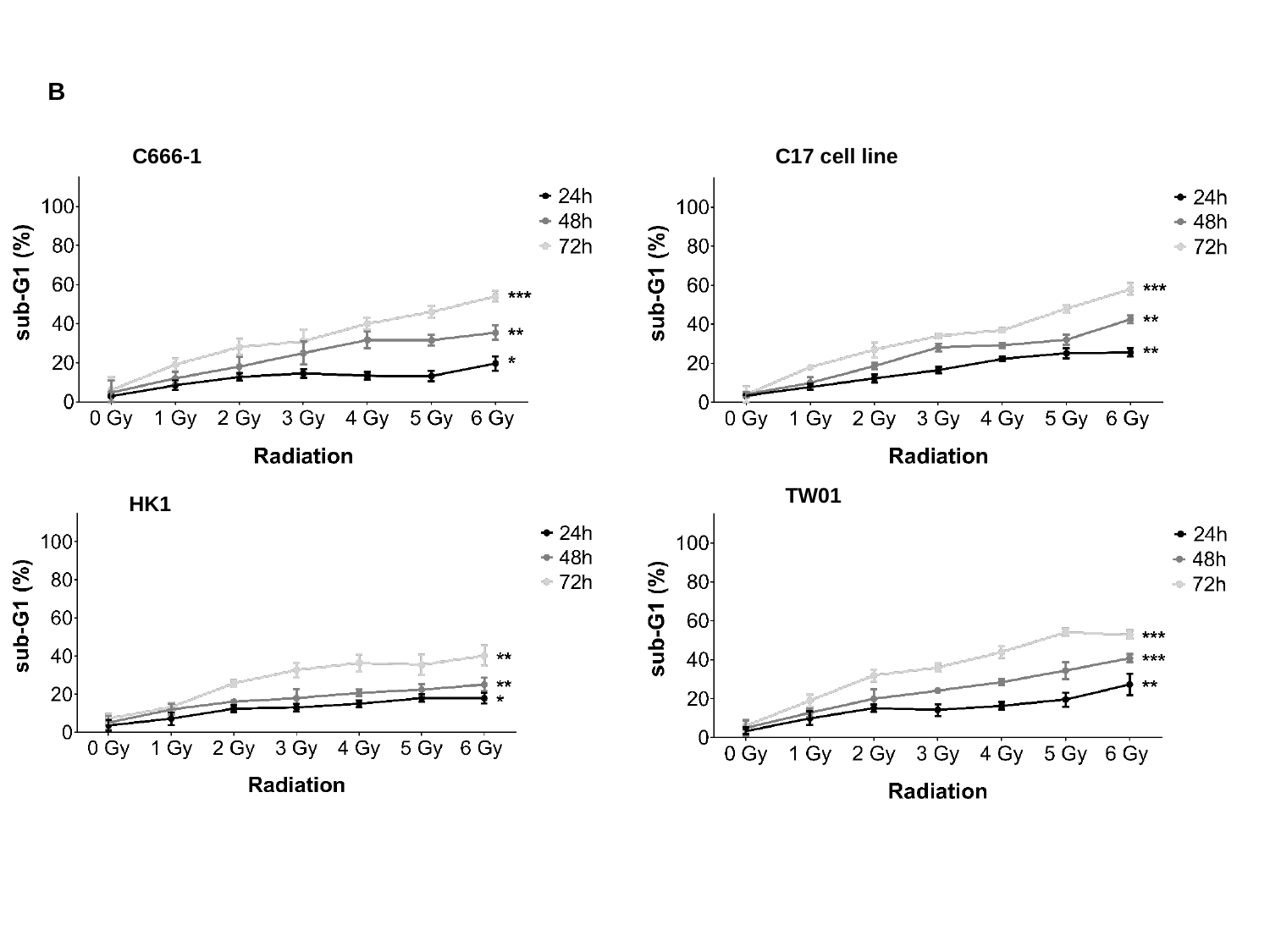

B
C17 cell line
C666-1
TW01
HK1
