# Supplementary material for: Radiotherapy Combined with PD-1 Inhibition Increases NK Cell Cytotoxicity towards Nasopharyngeal Carcinoma Cells
Source: Cells. 2021 Sep 17;10(9):2458. doi: 10.3390/cells10092458 (PMC8470143; doi:10.3390/cells10092458)
Supplement: Supplementary file 1 [file cells-10-02458-s001.zip › Supplementary figure 2.pptx]

## Slide 1
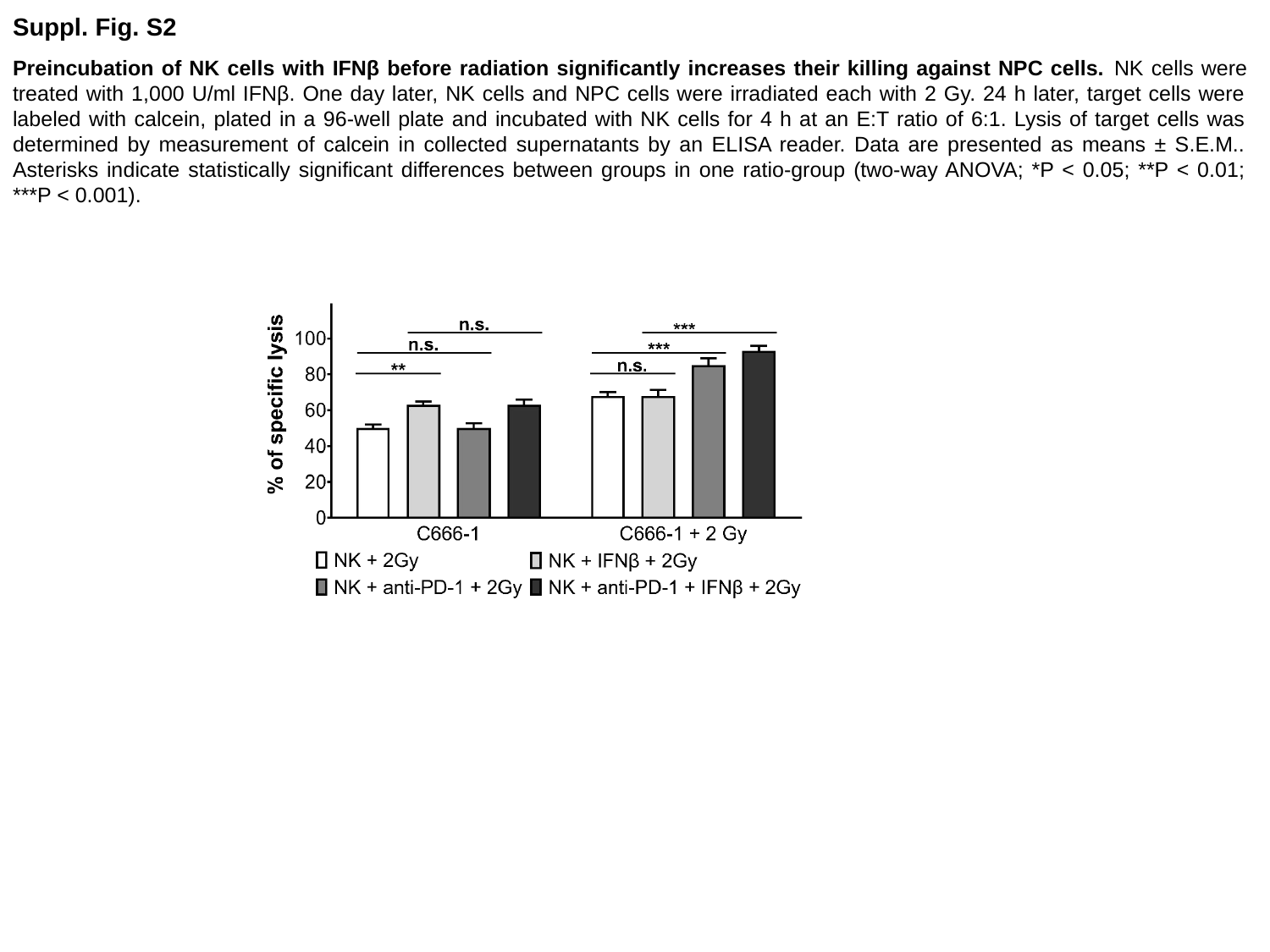

Suppl. Fig. S2
Preincubation of NK cells with IFNβ before radiation significantly increases their killing against NPC cells. NK cells were treated with 1,000 U/ml IFNβ. One day later, NK cells and NPC cells were irradiated each with 2 Gy. 24 h later, target cells were labeled with calcein, plated in a 96-well plate and incubated with NK cells for 4 h at an E:T ratio of 6:1. Lysis of target cells was determined by measurement of calcein in collected supernatants by an ELISA reader. Data are presented as means ± S.E.M.. Asterisks indicate statistically significant differences between groups in one ratio-group (two-way ANOVA; *P < 0.05; **P < 0.01; ***P < 0.001).
