# Supplementary material for: Radiotherapy Combined with PD-1 Inhibition Increases NK Cell Cytotoxicity towards Nasopharyngeal Carcinoma Cells
Source: Cells. 2021 Sep 17;10(9):2458. doi: 10.3390/cells10092458 (PMC8470143; doi:10.3390/cells10092458)
Supplement: Supplementary file 1 [file cells-10-02458-s001.zip › Supplementary figure 3.pptx]

## Slide 1
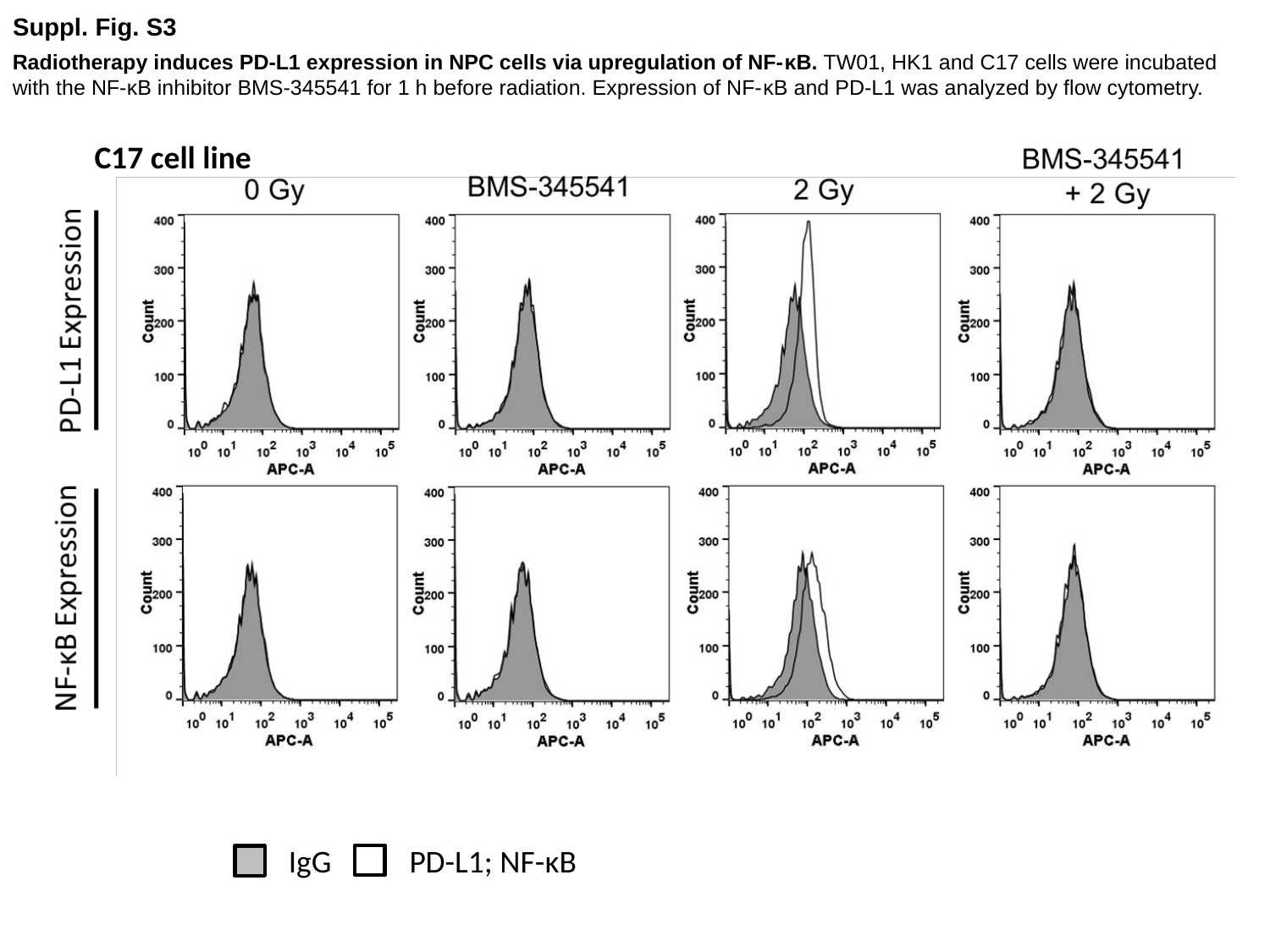

Suppl. Fig. S3
Radiotherapy induces PD-L1 expression in NPC cells via upregulation of NF-κB. TW01, HK1 and C17 cells were incubated with the NF-κB inhibitor BMS-345541 for 1 h before radiation. Expression of NF-κB and PD-L1 was analyzed by flow cytometry.
C17 cell line
PD-L1; NF-κB
IgG

## Slide 2
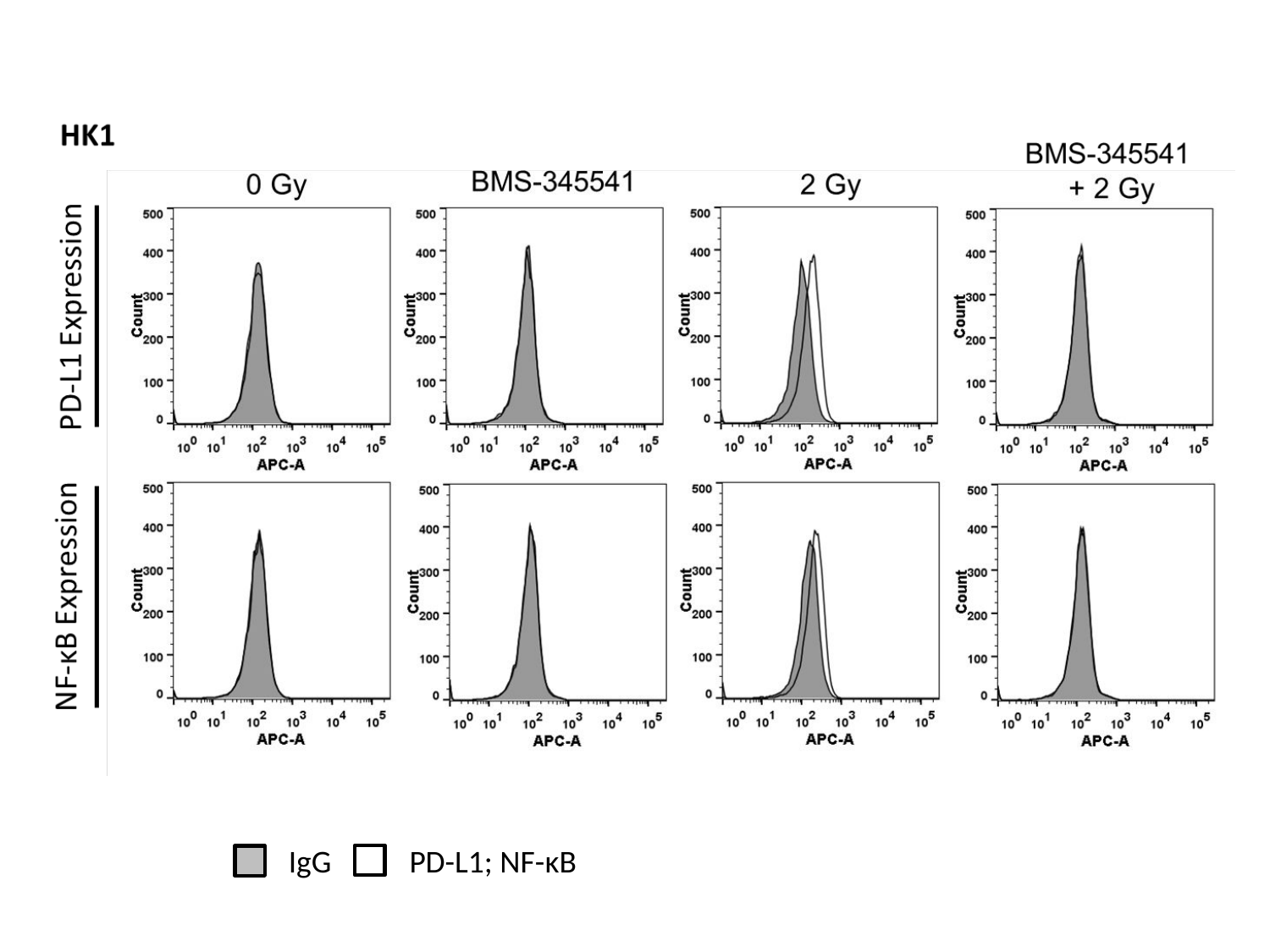

PD-L1; NF-κB
IgG

## Slide 3
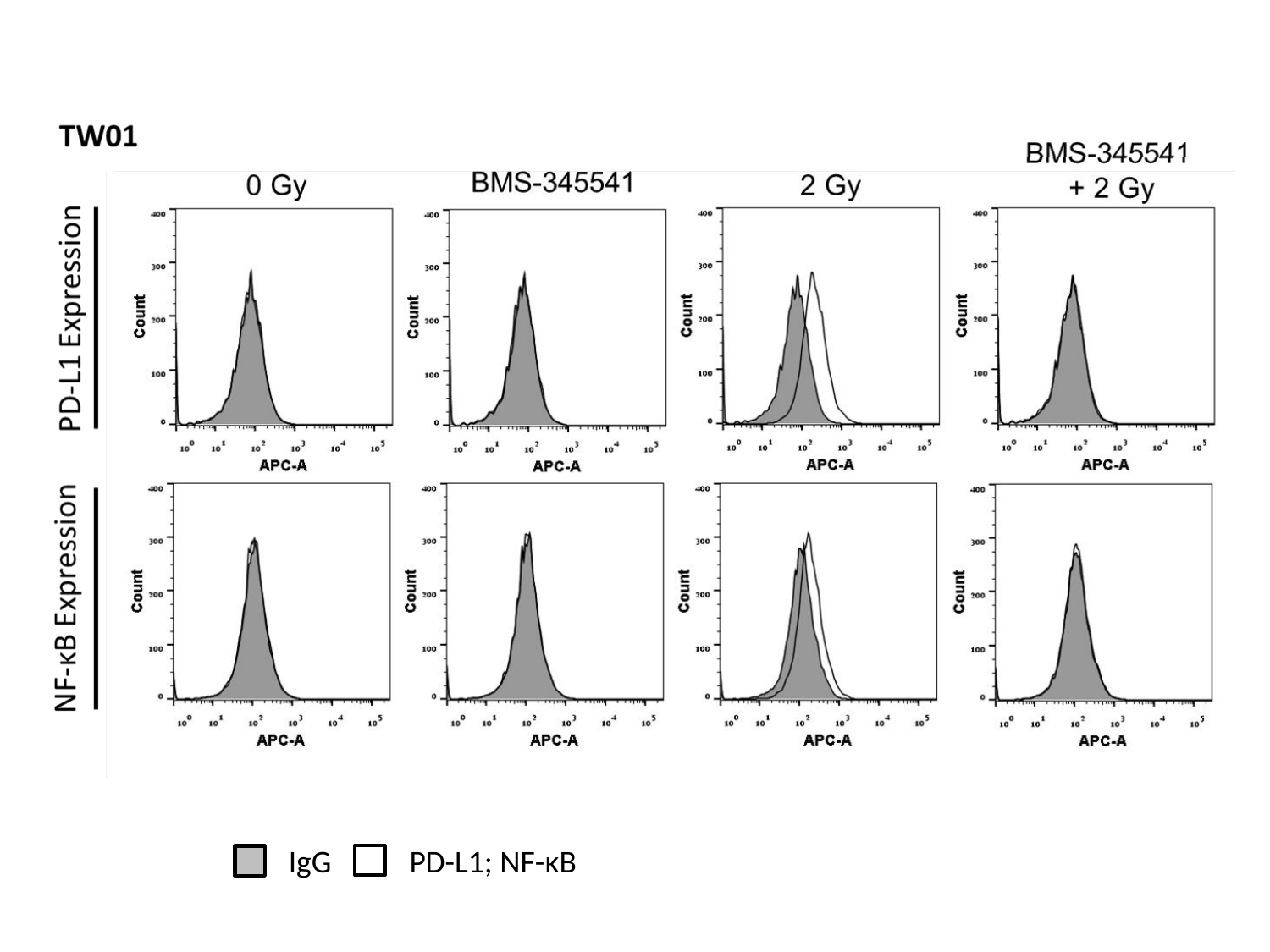

PD-L1; NF-κB
IgG
